# Blubber transcriptome response to acute stress axis activation involves transient changes in adipogenesis and lipolysis in a fasting-adapted marine mammal

**DOI:** 10.1038/srep42110

**Published:** 2017-02-10

**Authors:** J. I. Khudyakov, C. D. Champagne, L. M. Meneghetti, D. E. Crocker

**Affiliations:** 1 Department of Biological Sciences, University of the Pacific, 3601 Pacific Avenue, Stockton, CA 95211, USA; 2 National Marine Mammal Foundation, 2240 Shelter Island Drive Suite 200, San Diego, CA 92106, USA; 3 Department of Biological Sciences, Old Dominion University, 1 Monarch Way, Norfolk, VA 23529, USA; 4 Department of Biology, Sonoma State University, 1801 E Cotati Avenue, Rohnert Park, CA 94928, USA

## Abstract

Stress can compromise an animal’s ability to conserve metabolic stores and participate in energy-demanding activities that are critical for fitness. Understanding how wild animals, especially those already experiencing physiological extremes (e.g. fasting), regulate stress responses is critical for evaluating the impacts of anthropogenic disturbance on physiology and fitness, key challenges for conservation. However, studies of stress in wildlife are often limited to baseline endocrine measurements and few have investigated stress effects in fasting-adapted species. We examined downstream molecular consequences of hypothalamic-pituitary-adrenal (HPA) axis activation by exogenous adrenocorticotropic hormone (ACTH) in blubber of northern elephant seals due to the ease of blubber sampling and its key role in metabolic regulation in marine mammals. We report the first phocid blubber transcriptome produced by RNAseq, containing over 140,000 annotated transcripts, including metabolic and adipocytokine genes of interest. The acute response of blubber to stress axis activation, measured 2 hours after ACTH administration, involved highly specific, transient (lasting <24 hours) induction of gene networks that promote lipolysis and adipogenesis in mammalian adipocytes. Differentially expressed genes included key adipogenesis factors which can be used as blubber-specific markers of acute stress in marine mammals of concern for which sampling of other tissues is not possible.

Stress can compromise an animal’s fitness via wide-ranging effects on metabolism, growth, immune capacity, and reproduction, which can ultimately impact the stability of populations^1^. The effects of stress on energy balance may be especially deleterious to animals that undergo fasting as part of their natural life histories^2^. For example, due to competing requirements of terrestrial breeding and aquatic foraging, phocid seals exhibit some of the most dramatic fasting adaptations known in mammals. Northern elephant seals (*Mirounga angustirostris*) undergo terrestrial fasts of up to four months that are coupled with energy-intensive activities such as breeding, lactation, molting and post-weaning development^3^. Fasting metabolism in phocids is fueled by large adipose stores (blubber) accrued during foraging, which are mobilized in response to insulin suppression and elevation of the glucocorticoid (GC) cortisol across the fast^4^.

GCs (cortisol, corticosterone) function as part of the hypothalamic-pituitary-adrenal (HPA) stress axis to regulate metabolic substrate availability during both rest and duress. In response to acute stress, adrenocorticotropic hormone (ACTH) released by the pituitary stimulates GC synthesis and release by the adrenal glands. GCs promote catabolism of nutrient stores and suppress energy-intensive processes such as reproduction, growth, and immune function^5^. While acute stress responses are tightly regulated by negative feedback, repeated or sustained stress may impair this regulation and impact an animal’s ability to conserve metabolic stores and participate in activities that require high energy expenditure (i.e. reproduction)^1^. Animals are becoming increasingly exposed to sources of anthropogenic disturbance that may cause stress and have adverse physiological impacts. For example, marine mammals may experience frequent acoustic disturbance (sonar, oil drilling, boat traffic), negative interactions with fisheries, and disturbance by coastal development^6^. Understanding how free-ranging animals, especially those already experiencing physiological extremes, regulate acute stress responses is critical for evaluating the impacts of chronic stress on physiology and fitness in wildlife in order to inform conservation management decisions. Due to the practical limitations of field sampling, however, many studies of stress in free-ranging animals have been limited to baseline hormone measurements^1^.

GCs exert pleiotropic physiological effects by directly influencing gene expression in target tissues such as adipose, muscle, and liver. GCs bind to intracellular receptors (glucocorticoid receptor, GR, and mineralocorticoid receptor, MR), which translocate to the nucleus, bind to response elements (e.g. glucocorticoid response elements, or GREs) of target genes, and alter gene expression. GR target genes may comprise more than 10% of the genome and include those involved in metabolism, immunity, and cell survival and growth, among others^7^. For example, in white adipose tissue (WAT) of terrestrial mammals, GCs increase fatty acid availability and adipocyte differentiation by upregulating expression of lipases and pro-adipogenesis factors (e.g. peroxisome proliferator-activated receptor, PPAR, and CCAAT/enhancer-binding protein, CEBP)^8^,^9^.

Other components of the HPA axis may also regulate adipose physiology. In addition to GCs, ACTH also stimulates synthesis and release of the MR-specific ligand aldosterone^10^, a crucial component of the stress response in marine mammals^11^ that may play a role adipogenesis^12^. There is recent evidence that MR is expressed at higher levels than GR in adipose, and may mediate the majority of GC effects in adipose tissue^13^. Some of these effects may occur through non-genomic pathways via membrane-bound MR^14^. In addition, ACTH receptors (MC2R) are also expressed at low levels in adipocytes and have been shown to stimulate lipolysis *in vitro*^15^. Therefore, transcriptional responses of adipose tissue to HPA axis activation are mediated by at least 3 hormones – cortisol, aldosterone, and ACTH – acting via multiple hormone receptors.

While gene expression changes can supplement hormone measurements as markers of stress, few studies have examined the downstream molecular effects of acute HPA axis activation in wild animals. We recently used an RNA sequencing (RNAseq) approach to identify genes differentially expressed in skeletal muscle of free-ranging elephant seals in response to acute corticosteroid elevation induced by administration of exogenous ACTH (exACTH)^16^,^17^. Here, we profiled the transcriptome response of elephant seal blubber to exACTH due to its ease of sampling and its key role in energy regulation, especially in marine mammals that include several species of conservation concern^18^. We produced the first phocid blubber transcriptome containing 140,672 annotated transcripts, which include many metabolic genes of interest, and identified key regulators of adipogenesis and lipid homeostasis as markers of acute HPA axis activation in blubber. These data represent a valuable addition to the conservation physiologist’s toolset for evaluating stress states in free-ranging marine mammal species of concern from which blubber, but not blood samples, are possible to obtain (e.g. cetaceans).

## Results

### Acute HPA axis stimulation

Acute corticosteroid elevation in juvenile northern elephant seals was induced by administration of exACTH, which has been shown to rapidly increase circulating cortisol, aldosterone, glucose, and free fatty acids in juveniles and adults of this species^16^,^19^,^20^. Four juvenile seals received an intramuscular injection of exACTH (mass-specific dose: 0.23 ± 0.02 U/kg; Table 1) to induce cortisol synthesis and release that would mimic a stress response to environmental factors (e.g. anthropogenic disturbance, predation). Blood and blubber tissue samples were collected at 3 time-points during the experiment: immediately after sedation (baseline) and 2 and 24 hours after exACTH administration (acute response and recovery, respectively). Due to the limitations of recapture, recovery samples were obtained for only two study subjects.

The initial (baseline) cortisol concentrations (124.6 ± 31.7 nM; Table 1) were similar to values previously reported for juvenile northern elephant seals^16^,^19^,^21^. Administration of exACTH significantly altered corticosteroid concentrations (cortisol: F_2,5_ = 260.80, p < 0.0001; aldosterone: F_2,5_ = 14.02, p < 0.01; Table 1). Both hormones increased (cortisol: 16-fold, aldosterone: 22-fold) in acute response samples collected 2 hours after exACTH administration (post-hoc least significant difference (LSD) tests, p < 0.01). The magnitude of this increase in corticosteroids was within the range that was elicited by handling stress without anesthesia in this species^22^. In the two recovery samples collected 24 hours after exACTH administration, both cortisol and aldosterone concentrations appear indistinguishable from baseline levels (Table 1), similarly to what was observed in a previous, comparable study^16^. Due to the small sample size of this study, the effect of exACTH on free fatty acid concentration (FFA) was not statistically significant (data not shown), although FFA weakly increased in response to exACTH in previous studies^16^,^19^,^20^.

### Blubber transcriptome assembly

Ten cDNA libraries (from 4 baseline, 4 acute response, and 2 recovery samples) were sequenced on one Illumina HiSeq 2500 lane, generating 32 Gbases of sequence data with 32.0 ± 2.8 million reads per sample. Raw reads were deposited at the NCBI Sequence Read Archive (SRA Accession: SRP045540; Samples: SAMN04595358–67). Raw sequences were quality-trimmed, abundance-normalized, and used for de novo Trinity assembly^23^. Trinity assembled 660.4 million sequenced bases into 510,060 contigs (or “transcripts”) in 391,055 “gene families.” The size of the assembly was 716 Mbases.

Assembly quality statistics obtained according to current recommendations^24^ are shown in Table 2. The overall TransRate quality score for the assembly was 0.42 (on a scale of 0 to 1), which is higher than >50% of the transcriptomes deposited in the NCBI Transcriptome Shotgun Assembly database^25^. Read mapping statistics were used to determine how accurately the assembly represented sequenced reads. Of the 27.33 million aligned reads, 87.32% mapped to the assembly as proper pairs, 8.91% mapped as improper pairs, and 1.90% and 1.88% mapped as left or right reads only. Transcriptome completeness was assessed by searching for single-copy orthologs of highly conserved vertebrate genes (Benchmarking Universal Single-Copy Orthologs, or BUSCOs^26^) encoded in the assembly. The blubber transcriptome contained 80% of the 3,023 BUSCOs in the Hierarchical Catalog of Orthologs (OrthoDB), of which 30% were duplicated in the assembly and 10% were fragmented copies. These metrics suggest that the transcriptome assembly is well-supported by the sequenced reads and contains the majority of highly conserved vertebrate genes.

Due high redundancy of the raw assembly, we filtered the transcriptome using TransDecoder, which retained transcripts with candidate protein coding regions while removing short and redundant reads^23^. The filtered assembly contained 49,345 genes, which represented 81% of BUSCOs, suggesting that filtering did not reduce transcriptome completeness. Both raw and filtered assemblies are available at https://figshare.com/s/69f88adad25c4093c602.

### Blubber transcriptome annotation

Vertebrate homologs of elephant seal transcripts and TransDecoder-predicted peptides were identified by BLASTX and BLASTP searches, respectively, of the UniProt/SwissProt proteome database with e-value cutoff of 10^−5^. BLASTX homologs were detected for 140,672 elephant seal transcripts. Of these, 24,804 elephant seal transcripts were matched by at least 10% of their length to a UniProt/SwissProt hit, 10,284 were matched by at least 90%, and 8,525 transcripts were matched by 100% of their length. BLASTP homologs were identified for 147,172 predicted elephant seal peptides. The Trinotate pipeline was used to identify additional functional annotation features: transmembrane protein regions (using TMHMM) and putative signaling peptides (using SIGNALP). Combined annotation results are available at https://figshare.com/s/69f88adad25c4093c602.

Taxonomic representation of top BLASTX hits to elephant seal transcripts included human (62.9% of total hits), cow (14.4%), mouse (9.9%), domestic dog (2.2%), rat (2.0%), orangutan (1.7%), pig (1.4%), slow loris (0.9%), and macaque (0.9%). Fifty northern elephant seal transcripts had best BLASTX hits to marine mammal sequences: 24 harbor seal (*Phoca vitulina*: RHO, MX1, MT-ND5, IL6, IL1B), 10 Weddell seal (*Leptonychotes weddellii*: HBB, APOA2, APOC1, LYZ), 4 grey seal (*Halichoerus grypus*: MB, LEP), 4 Atlantic bottlenose dolphin (*Tursiops truncatus*: CYBA), 4 Pacific walrus (*Odobenus rosmarus divergens*: APOA1, HBA), 1 common minke whale (*Balaenoptera acutorostrata*: CYP1A1), 1 southern elephant seal (*Mirounga leonina*: CYCS), 1 California sea lion (*Zalophus californianus*: APOE), and 1 northern fur seal (*Callorhinus ursinus*: NR1I3) sequences.

**Figure 1:**
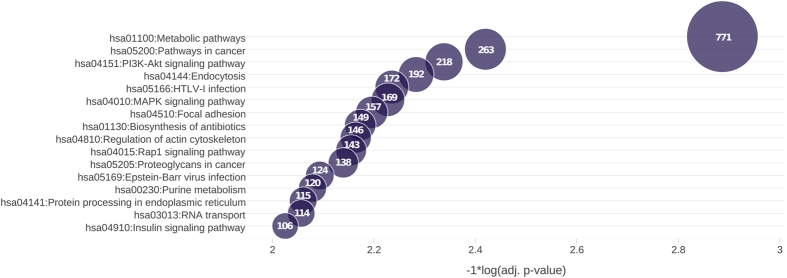
Top KEGG pathways significantly overrepresented in the elephant seal blubber transcriptome relative to the human proteome (p < 0.05). The x-axis shows significance of enrichment [−1 * log(p-value)]. Dot size is proportional to the number of elephant seal transcripts mapping to each overrepresented pathway.

Functional annotation was used to identify metabolic and signaling pathways that were enriched in the elephant seal blubber transcriptome relative to the human genome. The top 16 KEGG pathways significantly overrepresented (p < 0.05) in the blubber transcriptome are shown in Fig. 1. The most significantly enriched pathway was metabolism, with 771 genes mapping to this category. Other enriched pathways of interest not shown in Fig. 1 included fatty acid metabolism (40 genes) and adipocytokine (55 genes), mTOR (46 genes), AMPK (86 genes), FoxO (90 genes), thyroid hormone (80 genes) and NF-kappa B (61 genes) signaling pathways. Key adipocytokine pathway genes identified in the elephant seal blubber transcriptome included adiponectin and its receptors ADIPOR1 and ADIPOR2, PPARG coactivator 1 alpha, mTOR and PPARA, among others.

### Gene expression analysis

**Figure 2:**
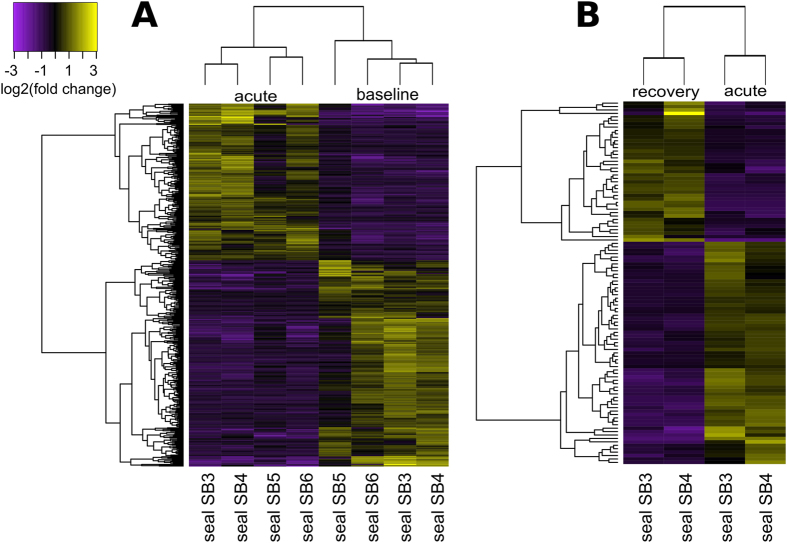
Differential gene expression during the acute response (acute/baseline; (**A**)) and recovery (recovery/acute; (**B**)) from exACTH administration. Heat maps depict gene expression changes (yellow: upregulated, purple: downregulated) between conditions, clustered by expression pattern.

**Figure 3:**
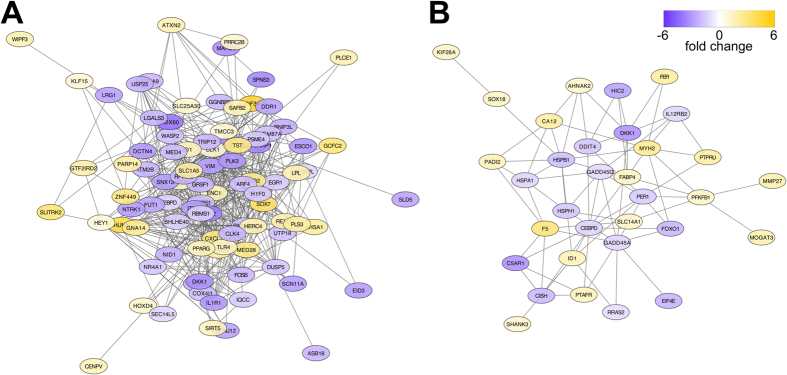
Predicted protein-protein interaction networks for genes differentially expressed during the acute response (acute/baseline; (**A**)) and recovery (recovery/acute; (**B**)) from exACTH administration. Nodes are color-coded by log2 fold-change in expression levels between conditions (yellow: upregulated, purple: downregulated). Network statistics are shown in Table 3.

In silico transcript abundance quantification was conducted using the ultrafast quasi-alignment tool kallisto, which pseudoaligned 94.45 ± 0.64% of sequencing reads from each sample to the assembly. Differential gene expression analysis was conducted at the gene level using DESeq2, selecting for genes with false discovery rate <0.05 and log2 fold-change >1. We identified 426 genes that were differentially expressed during the acute response to exACTH (acute/baseline), of which 184 were upregulated and 242 were downregulated in acute response relative to baseline samples (Fig. 2A, Supplementary File S1). Only 112 of these genes (46 upregulated, 66 downregulated) were annotated by BLASTX. We identified 106 genes differentially expressed during recovery from exACTH (recovery/acute), of which 41 were upregulated and 65 were downregulated in recovery relative to acute response samples (Fig. 2B, Supplementary File S2). Of these, only 46 differentially expressed transcripts (22 upregulated, 24 downregulated) were annotated. Lastly, we identified 313 genes that were differentially expressed during recovery relative to baseline (recovery/baseline), of which 141 were annotated (77 upregulated, 64 downregulated; Supplementary File S3). Annotated genes from the acute/baseline and recovery/acute datasets were used to predict protein-protein interaction networks based on interaction data from public databases using Cytoscape^27^ (Fig. 3). Network statistics^28^ shown in Table 3 suggest that differentially expressed genes likely function within transcriptional networks with high connectivity to orchestrate physiological responses to exACTH. The gene sets were not of sufficient size to obtain significant functional enrichment; therefore, differentially expressed genes were manually clustered into groups based on functional data from UniProt and literature search.

### Acute blubber transcriptome response to exACTH

The transcriptional signature of blubber during the acute response to exACTH suggested that both lipolysis and adipogenesis were increased (Supplementary File S1). Four key regulators of adipocyte differentiation (PPARG, CEBPD, KLF15, DKK1) were upregulated, while an inhibitor of differentiation (ID1) was downregulated. A key lipase (LPL), mitochondrial sirtuin (SIRT5), and beta-adrenergic receptor (ADRB2) were upregulated, while genes that antagonize lipolysis (LGALS3, EGR1) and promote insulin sensitivity (SORBS1, NR4A1) were downregulated. Two other genes recently implicated in control of fat mass and insulin sensitivity (TST, ATXN2) were upregulated. Five genes involved in proteolysis (PSME4, TRIP12, RFWD2, ASB18, USP25) were downregulated, but two others (HERC4, ENC1) were upregulated.

Blubber transcriptome response to exACTH reflected the pleiotropy of corticosteroid function and heterogeneity of the target cell population. Differentially expressed genes included those involved in immune signaling (up: FCGR2, CXCL11, TLR4; down: IRF2BPL, IL1R1, DCLK1, DDX60), cytoskeleton and extracellular matrix (ECM) remodeling (up: KIF3A, SLITRK2, PLS3, WIPF3; down: WASF2, NID1, CDH15, DDR1, DCTN4, VIM), and transcriptional regulation (up: GCFC2, ZNF449, MED28, GTF2IRD2, PARP14, SAFBP2, HOXD4, PRRC2B; down: DUSP5, MED4, FOSB, XRN2, EID3, MTERF1, GRSF1, SEC14L5). Genes involved in signal transduction (HUNK, GNA14, PLCE1, CLK1) and angiogenesis (SOX7, HEY1) were upregulated, while those associated with brown fat cell differentiation (LRG1), apoptosis (BNIP3L, NTRK1, ITPR1), and DNA replication (RBMS1, GINS4, ARPP19B, ESCO1, CDK5RAP1) were downregulated. Other upregulated genes of interest included a GR activity-enhancing chaperone (AHSA1) and a transporter of steroid hormones (SLC10A6). Other downregulated genes of interest included an inhibitor of clock genes (BHLHE40), factors associated with cell cycle and reactive oxygen species metabolic processes (ARF4, PLK3), and an epigenetic repressor of transcription (SUV420H1).

### Blubber transcriptome recovery from exACTH

Transcriptional recovery from acute corticosteroid elevation was examined by comparing expression values of recovery and acute response samples. Recovery from exACTH involved suppression of adipogenesis-inducing genes and upregulation of genes involved in lipid storage (Supplementary File S2). Pro-adipogenic factors that had been induced during the acute response (DKK1, CEBPD) were downregulated during recovery, while genes that promote cell proliferation and suppress differentiation (ID1, SOX18) were upregulated. Metabolic factors involved in triglyceride synthesis (MOGAT3), lipid import and storage (FABP4), carbohydrate transport (SLC35C1), and metabolic response to insulin, GCs, and fasting (PFKFB1) were upregulated during recovery as compared to the acute response. In contrast, genes involved in protein and lipid catabolism (DDIT4, FOXO1) and insulin signaling (CISH, EIF4E) were downregulated.

Other downregulated genes of interest included GR targets involved in cell stress responses and apoptosis (GADD45A, GADD45G), a clock gene (PER1), receptors for interleukin 12 (IL12RB2) and pro-inflammatory complement C5 (C5AR1), molecular chaperones (HSPA1, HSPH1), a signaling factor involved in osteoblast differentiation (RRAS), and general regulators of transcription (HIC2, ZNF462). Significantly, downregulation of the 70 kDa heat shock protein gene HSPA1, which interferes with GR ligand-binding activity^29^, suggested that recovery of sensitivity to GCs occurred within 24 hours. Other upregulated genes of interest included those associated with smooth muscle contraction (MYH2, PTARF, TPCN2), cell proliferation (FER, PTPRU), cytoskeleton (KIF26A), ECM remodeling (MMP27), and ion transport (CA12, SLC26A10, SLC14A1).

Expression values in recovery samples were also compared to baseline to identify genes that remained up- or downregulated after corticosteroid levels returned to baseline levels (Supplementary File S3). Fourteen genes were identified in both recovery/baseline and recovery/acute datasets (AHNAK2, C5AR1, DDIT4, EIF4E, FOXO1, GADD45A, HIC2, HSPA1, HSPH1, MOGAT3, PER1, PIGW, RRAS, SLC14A1). Upregulated genes that were uniquely identified in the recovery/baseline dataset included those involved in fatty acid biosynthesis (ACSM1) and regulation of mTOR activity (WDR59), insulin signaling (FAMD3), and heat shock response (HSPA8). Downregulated genes included those associated with proteolysis (SPSB1, CAST, USP25), circadian clock (CRY), apoptosis (IVNS1ABP, RHOB, CHAC), and cellular response to GCs (KLF9). Other genes of interest included factors involved in development and differentiation (up: TBX3, SOX21, HOXB4, HOXD4; down: CHSY1, NOV, ATOH8, PTCH2, LGALS3), immune and inflammatory signaling (up: CCR1, CCR5, PARP4, ILRL1, CD4, KLHL6, KIF3A, TRAF2, TNFSF10, P2RX7), G protein-coupled and GTPase signaling (up: GPBAR1, GIMAP7, RASGEF1B, TBC1D2B, SH3PXD2A; down: ASAP3, INPP5B), general regulation of transcription (up: ZNF37A, ZNF263, BANP, TRIP4, NFYA; down: XRN2, ZNF383, ZNF451, MED4), and ECM remodeling and cell-cell adhesion (up: LAMB4; down: COL4A2, VIM, LOXL2, FBN1).

### Validation of candidate markers

**Figure 4:**
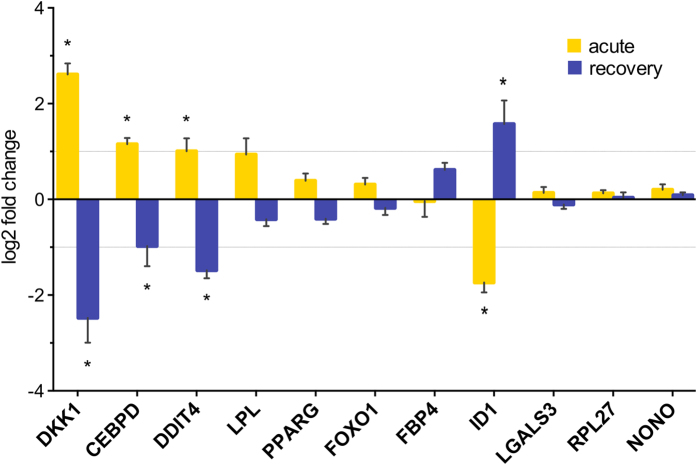
Log2-fold change values determined by qPCR for 11 markers identified as differentially expressed in the transcriptome. Error bars depict standard error of the mean. *Denotes delta C_T_ values that were significantly different between conditions (p < 0.05). Dotted line indicates log2 fold-change threshold of 1.0.

Due to the small sample size of the RNAseq dataset, differential gene expression results were validated by qPCR in sequenced samples and additional blubber samples collected from five animals during an identical experimental protocol in 2013^16^ (for a total of 9 animals; Table 1). There were no differences in corticosteroid profiles between the two experiments (cortisol: p = 0.9639, aldosterone: p = 0.2527). Twelve candidate genes for qPCR were chosen based on biological functions of interest, transcript abundance in sequenced samples (average transcripts per million (TPM) >3), and change in expression between conditions (upregulated, downregulated, no change). These included DKK1, CEBPD, DDIT4, LPL, PPARG, FOXO1, FABP4, ID1, and LGALS3, and candidate reference genes RPL27, NONO, and YWHAZ (Table 4). YWHAZ was selected as the optimal reference gene based on stability of expression across samples (Pearson r = 0.976, p < 0.001). Log2-transformed fold-change values determined by RNAseq and qPCR were significantly correlated (Pearson r = 0.83, p < 0.0001). Expression of markers did not vary by sex (p > 0.05). All markers that were upregulated during acute response were downregulated during recovery and vice versa, confirming that transcriptional response to exACTH was highly specific (Fig. 4). DKK1, CEBPD, and DDIT4 were significantly upregulated during the acute response to exACTH and downregulated during recovery (DKK1: F_2,15_ = 23.42, p < 0.0001; CEBPD: F_2,15_ = 8.54, p = 0.0035; DDIT4: F_2,14_ = 19.77, p < 0.0001). While not statistically significant, LPL, PPARG, and FOXO1 showed a similar trend. In contrast, ID1 was significantly downregulated during the acute response and upregulated during recovery (F_2,15_ = 19.94, p < 0.0001), and FABP4 showed a similar trend. Differences in magnitude of fold-change values were likely due to differences in detection sensitivity of the two methods, with RNAseq being considerably more sensitive than qPCR^30^. Overall, expression data obtained by the two techniques was well-correlated and showed that DKK1, CEBPD, DDIT, and ID1 may be robust qPCR markers of acute HPA axis activation in blubber.

## Discussion

The goals of this study were to profile the transcriptome response of blubber to HPA axis activation in fasting-adapted species and identify tissue-specific molecular markers of stress in marine mammals. The transcriptome was produced by RNA sequencing of blubber samples collected prior to, during, and following administration of a single dose of exACTH to juvenile elephant seals, which induced acute elevation in endogenous corticosteroids that lasted <24 hours, similar to previous experiments^16^,^19^,^20^. The transcriptome assembly contained thousands of transcripts with homology to mammalian genes involved in lipid homeostasis, adipokine signaling, and other functions of interest. Genes differentially expressed during acute response to exACTH included transcriptional regulators of adipogenesis and lipid metabolism, while recovery from exACTH involved their rapid suppression and upregulation of genes involved in lipid synthesis and storage.

While cellular responses to corticosteroids have been examined extensively in laboratory and disease models and *in vitro* study systems, little is known about the coordinated responses of complex tissues to HPA axis activation, especially in free-living animals. Here, we profiled the transcriptome of elephant seal blubber tissue due to its accessibility in marine mammals and its key role in metabolic homeostasis, especially in fasting-adapted species. In marine mammal species of concern such as large cetaceans, blubber may be the only tissue that is possible to obtain (via biopsy dart) to evaluate stress states and its impacts^18^. Marine mammal blubber is a multi-functional tissue that is stratified into at least two morphologically and functionally distinct layers. A dense outer layer performs thermoregulatory and structural roles, while a highly vascularized, metabolically active inner layer is used for lipid storage and mobilization according to energy demands^31^. The inner stratum, which was used in this study, contains a heterogeneous mix of white adipocytes, brown adipocytes, connective tissue, cutaneous muscle, and nerve fibers^32^. While detailed cellular analysis of phocid blubber has not been conducted, it probably contains other cell types also found in white adipose tissue (WAT) of terrestrial mammals: mesenchymal stem cells, fibroblasts, endothelial cells, macrophages, and preadipocytes in various stages of commitment^33^. Therefore, the blubber transcriptome represents coordinated transcriptional activity of diverse cell types.

The transcriptome was assembled de novo using Trinity, producing 510,060 contigs, of which 140,672 shared sequence similarity with known vertebrate proteins. These included members of metabolic and adipocytokine signaling pathways of interest to comparative physiology studies. Assembly quality was evaluated using several metrics of assembly accuracy and completeness^24^: (1) mapping rate of sequenced reads, (2) TransRate score, and 3) BUSCO analysis. The blubber transcriptome assembly had a proper read pair mapping rate of 87.32%, a TransRate score of 0.42 (a relatively high rating^25^), and contained 80% of highly conserved vertebrate orthologs. However, the annotation rate (27.6% of transcripts annotated) for blubber was lower than that of elephant seal muscle (66.1% of transcripts)^17^, which may be the result of differences in annotation methods used and the poor representation of blubber tissue in reference databases. Of the top BLASTX hits to elephant seal transcripts, the second most highly represented species after human was bovine (*Bos taurus*, 14.4% of annotated transcripts). This was surprising as pinnipeds are more closely related to canids than bovines^34^. However, high representation of bovine homologs in the seal transcriptome may be an artefact of genome annotation quality rather than phylogeny, as the Bovine Genome Database is curated by a large-scale collaborative effort^35^. Further phylogenomic analyses will be necessary to address this hypothesis.

We identified 426 and 112 genes that were differentially expressed during the acute response and recovery from exACTH, respectively. In addition, 313 genes were differentially expressed between recovery and baseline conditions. Due to annotation challenges described above, only 26% of the acute response genes and 45% of recovery genes were annotated, limiting our downstream analyses to highly conserved genes overrepresented in sequenced genomes of terrestrial mammals. Differentially expressed genes included corticosteroid receptor targets involved in regulation of adipogenesis and lipid homeostasis, including several that were identified in elephant seal muscle during a similar exACTH experiment^16^.

The acute metabolic response of elephant seals to exACTH was characterized by differential expression of genes that promote lipid catabolism and oxidation at the expense of synthesis and storage, a characteristic response to GCs^9^. However, significant changes in circulating free fatty acids were not observed 2 hours after exACTH administration; appreciable changes in circulating metabolites may not be manifested as quickly as changes in gene expression. Transcriptional changes during the acute response to exACTH included upregulation of lipoprotein lipase (LPL), a key regulator of fatty acid uptake and availability^36^, mitochondrial sirtuin SIRT5, which promotes beta-oxidation of fatty acids^37^, and the pro-lipolytic catecholamine receptor ADR2B^38^, concomitant with downregulation of galectin-3 (LGALS3)^39^ and early growth response protein 1 (EGR1)^40^, both of which inhibit the rate-limiting enzyme in lipolysis (adipose triglyceride lipase, ATGL)^41^. Maintenance of insulin resistance, characteristic of fasting elephant seals^42^, was evidenced by downregulation of orphan receptor NR4A1 (Nur77)^43^ and sorbin and SH3 domain containing 1 (SORBS1)^44^, which promote insulin sensitivity. In contrast to this study, SORBS1 was upregulated in elephant seal skeletal muscle in response to exACTH^16^, suggesting that its role in corticosteroid responses may be tissue-specific. Two genes recently implicated in control of insulin sensitivity and fat mass in obese humans – mitochondrial thiosulfate sulfurtransferase (TST)^45^ and ataxin-2 (ATXN2)^46^ – were upregulated. Their functions in other contexts are currently unknown.

Metabolic recovery from exACTH administration was detected within 24 hours of administration as upregulation of genes that facilitate lipid synthesis (monoacylglycerol O-acyltransferase 3, MOGAT3) and uptake (fatty acid binding protein 4, FABP4), and downregulation of genes involved in catabolism (DNA damage-inducible transcript 4 DDIT4/REDD1 and forkhead box protein FOXO1)^47^. In addition to its well-described role in proteolysis, FOXO1 also promotes lipolysis by regulating ATGL expression^48^. Both DDIT4 and FOXO1 were also downregulated in elephant seal muscle during recovery from exACTH^16^, suggesting that corticosteroid-induced catabolism of nutrient stores is generally tightly controlled in a fasting-adapted mammal.

Paradoxically, corticosteroids simultaneously promote lipolysis and adipogenesis in mammalian WAT. Recent studies suggest that the latter may be effected through MR activation by GCs and aldosterone^13^. Adipogenesis – the process by which pre-adipocyte precursors residing within adipose tissue differentiate into mature functional adipocytes – is positively regulated by PPARG, CCAAT-enhancer-binding proteins (C/EBP) and Kruppel-like transcription factors (KLF) and opposed by Wnt signaling and ID transcription factors^33^. The blubber transcriptome response to exACTH was consistent with transient induction of an adipogenesis programme. PPARG, CEBPD, KLF15, and the Wnt inhibitor DKK1 were upregulated, while ID1 was strongly downregulated. CEBPD and KLF15 were also upregulated in elephant seal skeletal muscle in response to exACTH^16^, consistent with their complex roles in both lipid and protein homeostasis^49^,^50^. The effects of exACTH on adipogenesis were transient: during recovery the pro-adipogenic factors were suppressed, while genes known to promote proliferation of undifferentiated cells (ID1, SOX18) were upregulated. The role of corticosteroid-induced, post-embryonic adipogenesis is poorly understood, especially in non-model systems. We hypothesize that differentiation of pre-adipocyte progenitors to mature adipocytes capable of fatty acid uptake and re-esterification may enable animals to rapidly recycle lipid metabolites released during acute stress responses. This is especially critical for fasting animals, which rely on lipolysis and fatty acid re-esterification to maintain a constant energy supply during fasts that require high energy expenditure^4^.

Differential expression of genes involved in diverse processes such as immune signaling, angiogenesis, ECM remodeling, cell signaling, cell proliferation, and apoptosis in response to exACTH reflected the heterogeneity of adipose tissue and is beyond the scope of the current study. Genes of interest to further studies include clock genes (PER1, BHLHE40, CRY), which were involved in elephant seal transcriptome response to exACTH despite the lack of a diel pattern of corticosteroid release in this species^51^, and genes implicated in oxidative stress (ARF4, PLK3), which may be a deleterious consequence of HPA axis activation.

In summary, the elephant seal blubber transcriptome described here provides a valuable resource for further studies of adipose biology, energy homeostasis, and stress responses in fasting-adapted animals and marine mammals. We identified dozens of annotated genes and predicted gene networks that were differentially expressed in response to exACTH, some of which have not been previously examined in non-pathological contexts in free-ranging species. Finally, we isolated DKK1, CEBPD, DDIT4 and ID1 as potential markers of acute HPA activation in marine mammal blubber. These markers can be used as key tools for evaluating the effects of anthropogenic disturbance, such as ocean noise, on marine mammals of concern from which tissues other than blubber are challenging to obtain. Expression of the master energy regulators CEBPD and DDIT4 was also altered in elephant seal muscle in response to exACTH, suggesting that these markers may be used to evaluate stress states across multiple tissue matrices. Future work will be necessary to validate additional markers using an increased sample size and to determine if these markers, and whole-transcriptome responses in general, differ between acute and chronic stress states.

## Methods

### Study site and subjects

All experiments involving animals were performed in accordance with relevant guidelines and regulations. All animal handling procedures were approved by Sonoma State University and University of the Pacific Institutional Animal Care and Use Committees, Department of the Navy Bureau of Medicine and Surgery, and were conducted under National Marine Fisheries Service marine mammal permit 19108. Juvenile northern elephant seals (*Mirounga angustirostris*) were sampled at Año Nuevo State Reserve (San Mateo County, CA). Samples for RNAseq were obtained from 4 animals (3 females, 1 male) of similar age (~10 months), mass (134.3 ± 3.7 (SD) kg), and body condition in Oct.–Nov. 2014 (Table 1). Samples used for RNAseq validation by qPCR were collected from five animals (1 male, 5 female) in Oct. 2013 as described previously (Table 1)^16^.

### ExACTH administration and sampling

Study animals were chemically immobilized as previously described^16^. Baseline blood samples were obtained within a mean (±SD) of 15.7 ± 3.5 minutes of initial sedation and stored on ice until return to the laboratory. Blubber samples were collected from the posterior flank with a 6.0 mm diameter biopsy punch (Miltex, USA). The inner half of each biopsy sample was isolated, minced with a sterile scalpel, and placed in cryovials containing RNAlater (~300 mg tissue per 1.5 ml; Qiagen, USA). Samples were incubated for 24–48 hours at 4 °C, after which RNAlater solution was removed and samples were transferred to −80 °C for storage. After baseline sampling, each animal received an intramuscular injection of 30 U (mean (±SD) mass-specific dose: 0.23 ± 0.02 U/kg) corticotropin LA gel (Wedgewood Pharmacy, USA)^16^,^19^,^20^. Sedation was maintained and a second set of blood samples and biopsy (on the contralateral side of the animal) was collected 2 hours after administration to capture the acute response to exACTH. Animals were weighed, marked, and released to resume normal activity as described previously^16^. Two subjects were immobilized the following day, 26.4 and 30.6 hours after exACTH injection, and a third set of recovery blood and tissue samples was collected as described above.

### Hormone and metabolite assays

Blood samples were centrifuged at 5,000 rpm for 15 minutes at 4 °C and isolated serum samples were stored at −80 °C until processing. Serum cortisol and aldosterone concentrations were assayed in duplicate using I^125^ radioimmunoassays (MP Biomedicals, USA). The mean intra-assay coefficients of variation (CV%) for the duplicates were 1.6% and 2.5% for cortisol and aldosterone, respectively. The assay platform for both hormones was validated by demonstrating parallelism of diluted samples to the standard curve and spike recovery of standard additions of >97%. Free fatty acids were analyzed using an enzymatic fluorometric assay (Cayman, USA). Samples were analyzed in triplicate with mean intra-assay CV% of 3.7%.

### RNA isolation

Blubber samples were minced with a sterile scalpel on ice, added to Qiazol (~100 mg tissue per 1.0 ml; Qiagen, USA), and homogenized using 5-mm steel beads in a TissuseLyser instrument (Qiagen, USA; 2 cycles of 2 minutes at 20 mHz). Homogenates were further disrupted using a 21-gauge needle to shear genomic DNA, and centrifuged for 10 minutes to separate lipids and cellular debris. RNA was isolated from clean homogenates using Lipid RNeasy Tissue Kit (Qiagen, USA) with a 20-minute on-column DNase I digest (Qiagen, USA). Eluates were concentrated by sodium acetate precipitation. RNA concentration was estimated using Qubit fluorometer (Life Technologies, USA) and integrity was evaluated using 2100 Bioanalyzer (Agilent Technologies, USA). The mean (±SD) RIN for samples used for RNA sequencing was 8.04 ± 0.40 (lowest: 7.20, highest: 8.50). Bioanalyzer traces for each sequenced sample are shown in Supplementary File S4.

### Library preparation and RNA sequencing

cDNA library preparation and Illumina sequencing were performed at the University of California, Davis DNA Technologies Core Facility using standard protocols (TruSeq RNA Sample Prep Kit v2, Illumina, USA). Specifically, mRNA was isolated from total RNA samples using oligo-dT magnetic beads, fragmented, and used as template for first-strand cDNA synthesis with random hexamers (SuperScript II Reverse Transcriptase, Invitrogen, USA). After cDNA purification, overhang fragments were end-repaired. The 3′ ends of the blunt-ended fragments were adenylated and ligated to proprietary adapter oligonucleotides (Illumina, USA). Ligation products were amplified by 10–12 cycles of PCR before library quantification and validation (Agilent Technologies, USA). Individual libraries were barcoded and pooled. Sequencing was carried out for 100 cycles on the Illumina HiSeq 2500 platform, generating 32.012 total sequenced megabases and an average of 32.011 ± 2.81 million paired-end 100-bp reads per sample. Fastq files were generated using Illumina Casava pipeline v1.8.2. Raw reads were submitted to NCBI SRA (Accession: SRP045540; Samples: SAMN04595358–67).

### Transcriptome assembly

Transcriptome assembly was conducted using Amazon Web Services Elastic Cloud Compute service (AWS EC2) and Extreme Science and Engineering Discovery Environment (XSEDE)^52^. Sequenced read trimming and abundance normalization were conducted using khmer v0.8.4 mRNAseq protocol^53^. Specifically, Illumina sequencing adapters were trimmed from reads using Trimmomatic v0.30^54^. Sequences with quality scores ≤30 and quality base pair content ≤50% were removed using FASTX Toolkit v0.0.13.2. One round of digital normalization (diginorm^55^) was used to normalize coverage to 20X with k-mer threshold of 20. Assembly was conducted using normalized reads from all 10 samples using Trinity v2.0.6^23^ with default parameters (k-mer size of 25) and maximum memory size of 60 GB with 8 CPU. Assembly metrics were obtained using TransRate v1.0.1^25^. Mapping metrics for quality-trimmed reads were obtained using bowtie v1.1.1^56^. Transcriptome completeness was assessed using BUSCO v1.2^26^ (with BLAST + v2.3.0, HMMER v3.1b2 and EMBOSS v6.5.7; vertebrate BUSCO dataset downloaded 6/1/16).

### Transcriptome annotation

Candidate coding regions within transcript sequences were identified using TransDecoder v2.0.1^23^. The transcriptome was annotated using Trinotate v3.0.0^23^ pipeline. Sequence homologies between SwissProt reference proteomes and assembled transcripts and TransDecoder-predicted coding regions were identified using BLASTX and BLASTP, respectively, using DIAMOND v0.7.10^57^ and BLAST+ v2.3.0^58^. SwissProt and PFAM databases were downloaded on 3/15/16. Signal peptides and transmembrane domains were identified using SIGNALP v.4.1 and TMHMM v2.0, respectively, according to Trinotate protocol^23^. Results were compiled into an annotation report with e-value cutoff of 10^−5^. Functional annotation was conducted using DAVID Bioinformatics Resources v.6.8 (beta)^59^ with human genome as background. The software detected 24,351 DAVID IDs corresponding to human UniProt IDs, which were used as the input list.

### Gene expression analysis

Transcript abundance estimation was conducted using kallisto v0.42.4^60^. Differential expression analysis was conducted at the gene level using DESeq2^61^ (Bioconductor v3.2) with false discovery rate cutoff of 0.05 and log2 fold-change in expression cutoff of 1.0. Predicted protein-protein interaction networks based on differentially expressed genes were produced using GeneMANIA^62^ with network weighing based on input genes and no additional interacting partners or attributes shown. Predicted networks (non-directed) were analyzed using Cytoscape v3.4.0^27^. Network statistics were obtained using the NetworkAnalyzer tool in Cytoscape. Statistical parameters are defined in http://med.bioinf.mpi-inf.mpg.de/netanalyzer/help/2.7/index.html#simple 28.

### Quantitative PCR

cDNA was synthesized from 500 ng input of total RNA in 20-μl reactions using iScript Reverse Transcription Supermix (Bio-Rad, USA). cDNAs were diluted ten-fold and 2 μl were used in each 20-μl qPCR reaction with iTaq Universal SYBR Green Supermix (Bio-Rad, USA). Primers were designed using IDT PrimerQuest tool (sequences shown in Table 4). Primer sequence specificity was confirmed by BLASTN and potential for primer-dimer formation calculated using IDT OligoAnalyzer v3.1. All primers were used at 200 nM final concentration in each qPCR reaction. qPCR assays were conducted in triplicate on ABI PRISM 7000 Sequence Detection System (Applied Biosystems, USA) with standard deviation for technical replicates of <0.167 C_T_. No-template and no-reverse transcriptase controls were included in each run and showed no amplification. Primer specificity was determined by melt curve analysis and agarose gel electrophoresis of qPCR products. Primer efficiency was determined using standard curves with five 1:5 dilutions of cDNA^63^. Putative reference genes were selected based on stable transcript abundances across sequenced samples, and validated for expression stability by qPCR. YWHAZ was chosen as the most stable reference gene using RefFinder (http://fulxie.0fees.us/)^64^, which calculated a comprehensive stability ranking based on delta C_T_, BestKeeper, NormFinder, and GEnorm methods. Across all samples, the BestKeeper-calculated Pearson correlation coefficient of C_T_ for YWHAZ was 0.976, and NormFinder and GEnorm stability values were 0.126 and 0.338, respectively. Fold-change in gene expression between sampling conditions was calculated using the Pfaffl method^63^.

### Statistical analyses

Statistical analyses of hormone, metabolite, and qPCR data were conducted using JMP v12.1.0 (SAS, USA). Changes in hormone concentrations or delta C_T_ gene expression values in response to exACTH were analyzed using linear mixed models with sample group (baseline, acute response, and recovery) as a fixed effect and animal ID as a random effect. Response variables were log-transformed as necessary to meet distribution and variance assumptions. Post-hoc comparisons between repeated samples were conducted using Student’s t-test of LSD. Correlation between log2-transformed fold change values obtained by RNAseq and qPCR was determined using Pearson’s multivariate correlation analysis.

## Additional Information

**How to cite this article**: Khudyakov, J. I. *et al*. Blubber transcriptome response to acute stress axis activation involves transient changes in adipogenesis and lipolysis in a fasting-adapted marine mammal. *Sci. Rep.* **7**, 42110; doi: 10.1038/srep42110 (2017).

**Publisher's note:** Springer Nature remains neutral with regard to jurisdictional claims in published maps and institutional affiliations.

## Supplementary Material

Supplementary Dataset 1

Supplementary Dataset 2

Supplementary Dataset 3

Supplementary File S4

## Figures and Tables

**Table 1:** Sex, mass, exACTH dose, and serum corticosteroid values of juvenile elephant seals used in the study.

Seal ID	Date	Sex	Mass (kg)	ACTH (U/kg)	Cortisol (nM)			Aldosterone (pM)		
					Baseline	Acute	Recovery	Baseline	Acute	Recovery
Samples used for RNAseq										
SB3	11/7/14	F	130	0.25	102.4	2259.8*	98.8	1383.4	8553.6*	1176.1
SB4	11/7/14	F	139	0.23	167.2	1852.2*	133.1	586.0	5233.1*	894.9
SB5	11/28/14	M	134	0.21	105.0	1936.8*	NA	1784.6	4841.6*	NA
SB6	11/28/14	F	134	0.24	94.9	1905.2*	NA	1273.1	4077.1*	NA
Samples used for qPCR validation										
JS2	10/25/13	M	114	0.25	146.5	2759.0*	146.0	770.5	2582.3*	1423.2
JS4	11/9/13	F	118	0.24	192.0	2012.1*	146.0	914.3	4129.4*	584.8
JS5	11/9/13	F	133	0.21	185.7	1882.9*	431.3	972.0	4148.2*	2587.4
JS6	11/22/13	F	135	0.21	113.9	1305.7*	173.2	537.9	2547.7*	508.8
JS7	11/22/13	F	127	0.22	159.0	1516.1*	185.8	1287.0	4115.5*	1594.2

^*^Denotes values that were significantly different from baseline (p < 0.05).

**Table 2:** Northern elephant seal blubber transcriptome assembly metrics.

N reads per sample	32.0 ± 2.8 M
N assembled bases	660,425,775
N assembled transcripts	510,060
Assembly size	716 Mbases
Read pairs mapped to assembly	87.32%
TransRate score	0.4221
Mean transcript length	1,294.8 bp
Transcript N50	3,186 bp
Shortest transcript length	224 bp
Longest transcript length	36,322 bp
N BLASTX-annotated transcripts	140,672
Complete BUSCOs	80%
Duplicated BUSCOs	30%
Missing BUSCOs	8.6%

**Table 3:** Network statistics for predicted protein-protein interaction networks shown in Fig. 3.

Parameter	Acute/baseline	Recovery/acute	Range
number of nodes	93	33	>0
average number of neighbors	10.237	4.545	>0
network density	0.111	0.142	0–1
network centralization	0.220	0.148	0–1
network heterogeneity	0.579	0.534	0–1
network clustering coefficient	0.193	0.226	0–1

In highly connected networks, the density, centralization, heterogeneity, and clustering coefficients parameters are close to 1^28^.

**Table 4:** Candidate markers identified by RNAseq and validated by qPCR.

Transcript ID	Gene homolog	Protein name	RNAseq log2 FC		Primer sequence
			Acute	Recovery	
TR53098 c0_g1	DKK1	dickkopf-related protein 1	2.29 ± 0.39	−3.21 ± 0.40	F: CCAAGATCTGTAAACCTGTCCTC
					R: CACAGTAACAGCGCTGGAATA
TR63022 c0_g1	CEBPD	CCAAT/enhancer-binding protein delta	1.09 ± 0.30	−1.30 ± 0.27	F: CGACTTCAGCGCCTACAT
					R: CCTTGTGGTTGCTGTTGAAG
TR41227 c0_g1	LPL	lipoprotein lipase	1.85 ± 0.29	ND	F: CTCAGGGACACTGCTTCATAC
					R: GCTAAGAAAGACCACCTGAAGA
TR53922 c5_g1	PPARG	peroxisome proliferator-activated receptor gamma	1.68 ± 0.31	ND	F: GTGCAGCTATTGCAAGTCATAAA
					R: TGCGGACTTGTCTGCTAATAC
TR69848 c0_g1	ID1	DNA-binding protein inhibitor ID-1	−1.60 ± 0.39	1.64 ± 0.33	F: GATGACGTGCTGAAGGATCTC
					R: GTGCTGCTCTACGACATGAA
TR54987 c1_g2	LGALS3	galectin-3	−2.22 ± 0.37	ND	F: GTTGCCTGTCTTTCTTCCTTTC
					R: GGAATGATGTTGCTTTCCACTT
TR16531 c16_g1	DDIT4	DNA damage-inducible transcript 4 protein	ND	−1.76 ± 0.27	F: AGGAAGACTCGGCATACCT
					R: TGGCACACAAGTGCTCAT
TR68766 c9_g5	FOXO1	forkhead box protein O1	ND	−2.22 ± 0.47	F: CTGTTGCTGTCACCCTTATCT
					R: CCTCATCACCAAGGCCATC
TR40820 c3_g2	FABP4	fatty acid-binding protein, adipocyte	ND	1.42 ± 0.33	F: CAAAGCCCACTCCTACAGTT
					R: TGCTACCCTAATGGTTGAGATG
TR34357 c2_g1	RPL27	60 S ribosomal protein L27	ND	ND	F: CCTCTTGGCGATCTTCTTCTT
					R: TTGATGATGGCACCTCAGAC
TR6987 c5_g2	NONO	non-POU domain-containing octamer-binding protein	ND	ND	F: GAGGAAGGTTTCGGACTGTAAG
					R: GCGGAGATTGCCAAAGTAGA
TR33370 c1_g1	YWHAZ	14-3-3 protein zeta/delta	ND	ND	F: AGCAGAGAGCAAAGTCTTCTATT
					R: GACTGATCCACAATCCCTTTCT

RNAseq log2-transformed fold change (FC) values are presented ±standard error of the mean. ND: not detected, F: forward primer, R: reverse primer.
