# Supplementary figures and images for: Blubber transcriptome response to acute stress axis activation involves transient changes in adipogenesis and lipolysis in a fasting-adapted marine mammal
Source: Sci Rep. 2017 Feb 10;7:42110. doi: 10.1038/srep42110 (PMC5301240; doi:10.1038/srep42110)

**Supplementary File S4.** Capillary gel electrophoresis traces of RNA used for sequencing.

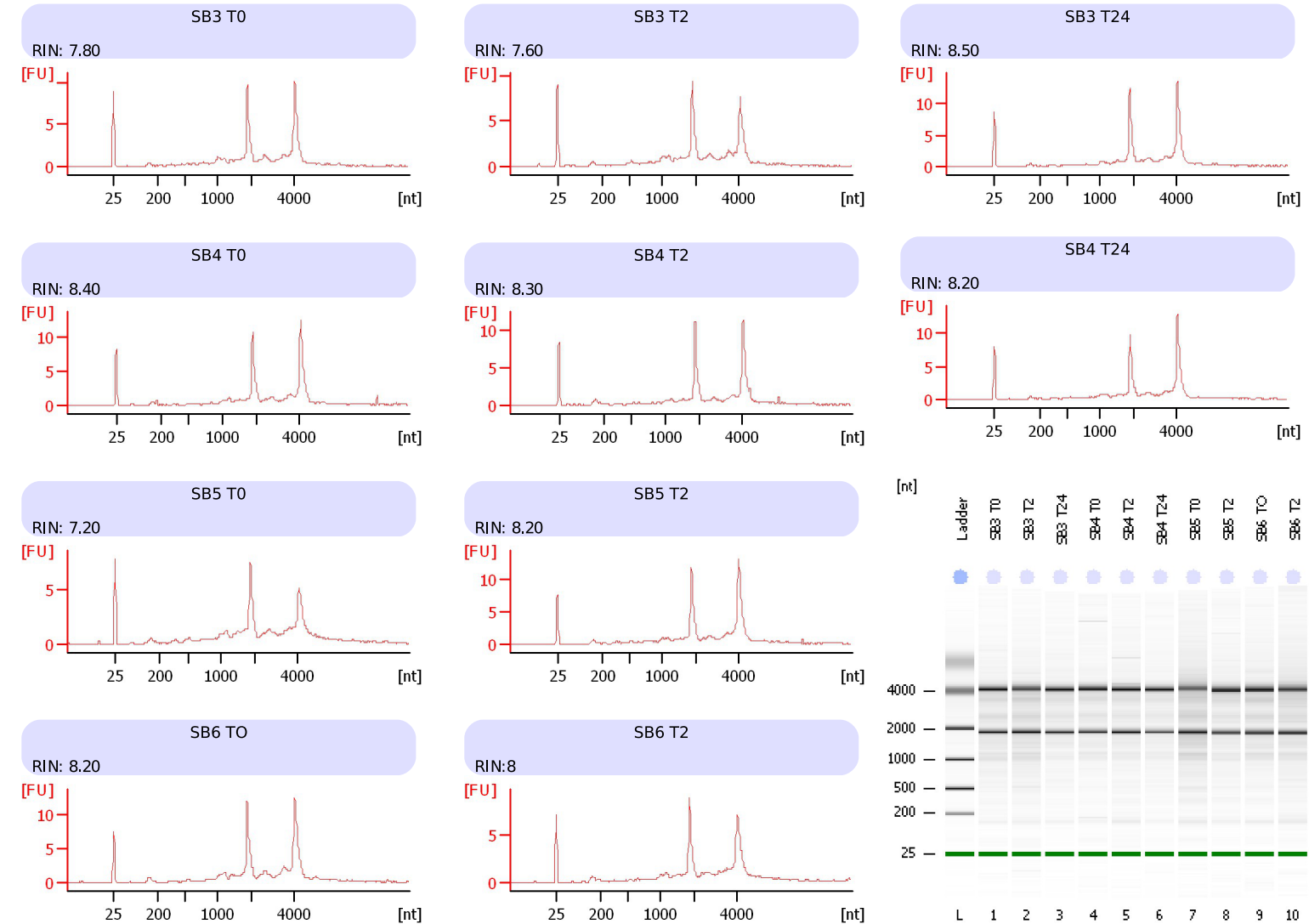

Supplement: Supplementary File S4 [file srep42110-s4.pdf]
